# Embolization of Carotid Body Tumors: Revisiting Direct Puncture Technique, Preliminary Experience and Literature Review

**DOI:** 10.7759/cureus.483

**Published:** 2016-02-07

**Authors:** Daniel R Felbaum, Hasan R Syed, Michael F McCullough, Rocco A Armonda, Ai-Hsi Liu, Randy S Bell

**Affiliations:** 1 Neurosurgery, Medstar Georgetown University Hospital, Washington DC, USA; 2 Neurointerventional Radiology, Medstar Washington Hospital Center, Washington DC, USA; 3 Department of Neurosurgery, Walter Reed Army Medical Center, Washington DC, USA

**Keywords:** carotid body tumors, direct puncture, onyx, tumor embolization

## Abstract

Preoperative embolization via transarterial route is an acceptable adjunct to the treatment of carotid body tumors (CBT). Direct tumor puncture for embolization has been previously described as a safe and feasible option. We revisit this technique and present our initial experience treating CBT via direct puncture.

We identified six patients that underwent preoperative embolization of CBT using a direct puncture technique embolized with Onyx (EV3 Micro Therapeutics Inc., Irvine, CA, USA). After defining the angioarchitecture via digital subtraction angiography, the tumor was targeted with Onyx. Using a 21-gauge needle, the tumor was punctured using a fluoroscopic road mask. There were no immediate post-procedural complications following embolization. All patients underwent definitive resection within 24 hours. During surgery, the embolization material did not affect surgical maneuvers. In addition, the estimated blood loss was noted to average 50 ml.

Although early in our experience, direct percutaneous embolization of CBT appears to be a reproducible and well-tolerated endovascular treatment option. Overall, the reported body of evidence available confirms the safety and efficacy of direct intralesional embolization with Onyx.

## Introduction

Endovascular therapy as an adjunct for treatment of vascular carotid body tumors (CBT) is well-established. The mainstay of therapy focuses on devascularization via the transarterial route, focusing mainly on the smaller branches of the external carotid artery. Although effective, the multiplicity and small caliber, highly tortuous arterial feeders make intervention more complex and time-consuming [1-2]. Earlier case reports employ n-BCA while more recent studies employ Onyx (EV3 Micro Therapeutics, Inc., Irvine, CA, USA) Employing intratumoral injection of an embolic agent has overall safely allowed near complete devascularization of these complex tumors [1-7].

## Case presentation

Methods

A retrospective chart review identified a series of six patients that had undergone direct intratumoral embolization therapy. Informed consent was obtained prior to any therapy. The series included five females and one male, and the average age of the patient was 62.6 years (range: 50-78). The average Shamblin score was 3.

Technique

Percutaneous or direct tumor puncture for intralesional embolization has been described in detail elsewhere [2, 8-9]. We have maintained this technique in our practice. In brief, a carotid occlusion test is initially performed in case of the need for the sacrifice of the carotid artery. Upon completion, the patient undergoes general anesthesia, is kept in the supine position with the head rotated 30 degrees towards the contralateral shoulder, and antibiotics are administered. Using digital subtraction angiography, the angioarchitecture of the carotid tumor is identified. Next, a 21-gauge needle is inserted in a safe distance within the lesion itself and confirmed with intralesional angiography. Slow, continuous backflow of blood through the needle confirms appropriate location. Afterward, Onyx-18 (ethylene vinyl alcohol copolymer) is injected under road mask fluoroscopy after standard preparation with dimethyl sulfoxide (DMSO). With tactile feedback, the intratumoral injection can be felt to have decreased penetration and repeat angiography can be performed to evaluate further treatment. If necessary, the spinal needle trajectory can be repositioned for further treatment.

Results

The mean fluoroscopy time was 39.5 minutes. The average amount of injected Onxy-34 was 2.98 ml. The extent of tumor devascularization was at least 70% in all cases, or a subtotal treatment session. Patients were monitored in surgical step-down unit with frequent neuro checks. There were no cases of airway compromise, inadvertent arterial embolization, or neurologic sequelae from the procedure.

Case illustration

**Figure 1:**
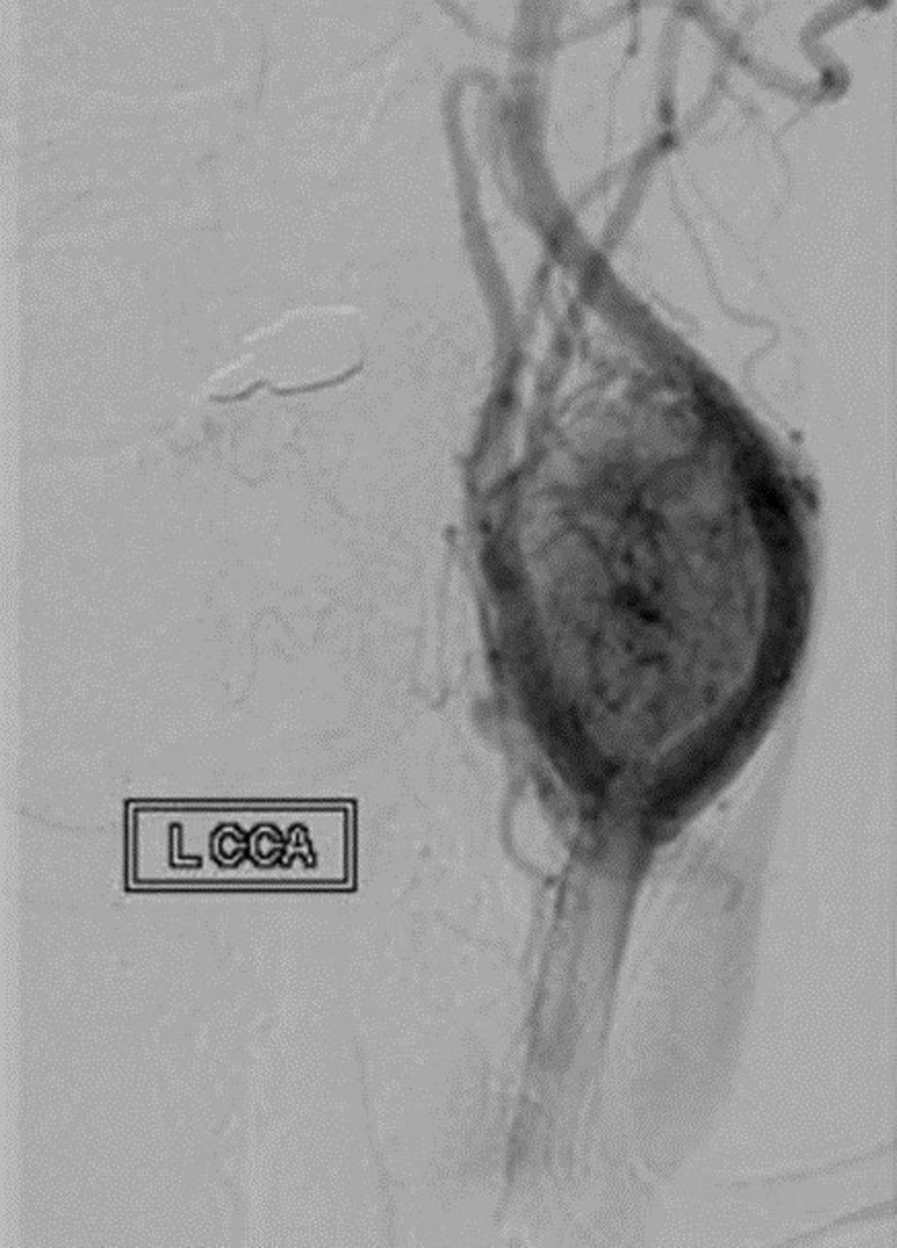
Digital subtraction angiography in AP projection revealing a vascularized carotid body lesion.

A 66-year-old right-handed female presented with a progressively enlarging left neck mass, consistent of a carotid body tumor. After a carotid balloon test occlusion was performed in the usual manner [10], the patient was intubated using general anesthesia. The left neck was anesthetized, prepped, and draped in the standard sterile manner. The lesion is defined in Figure 1.

**Figure 2:**
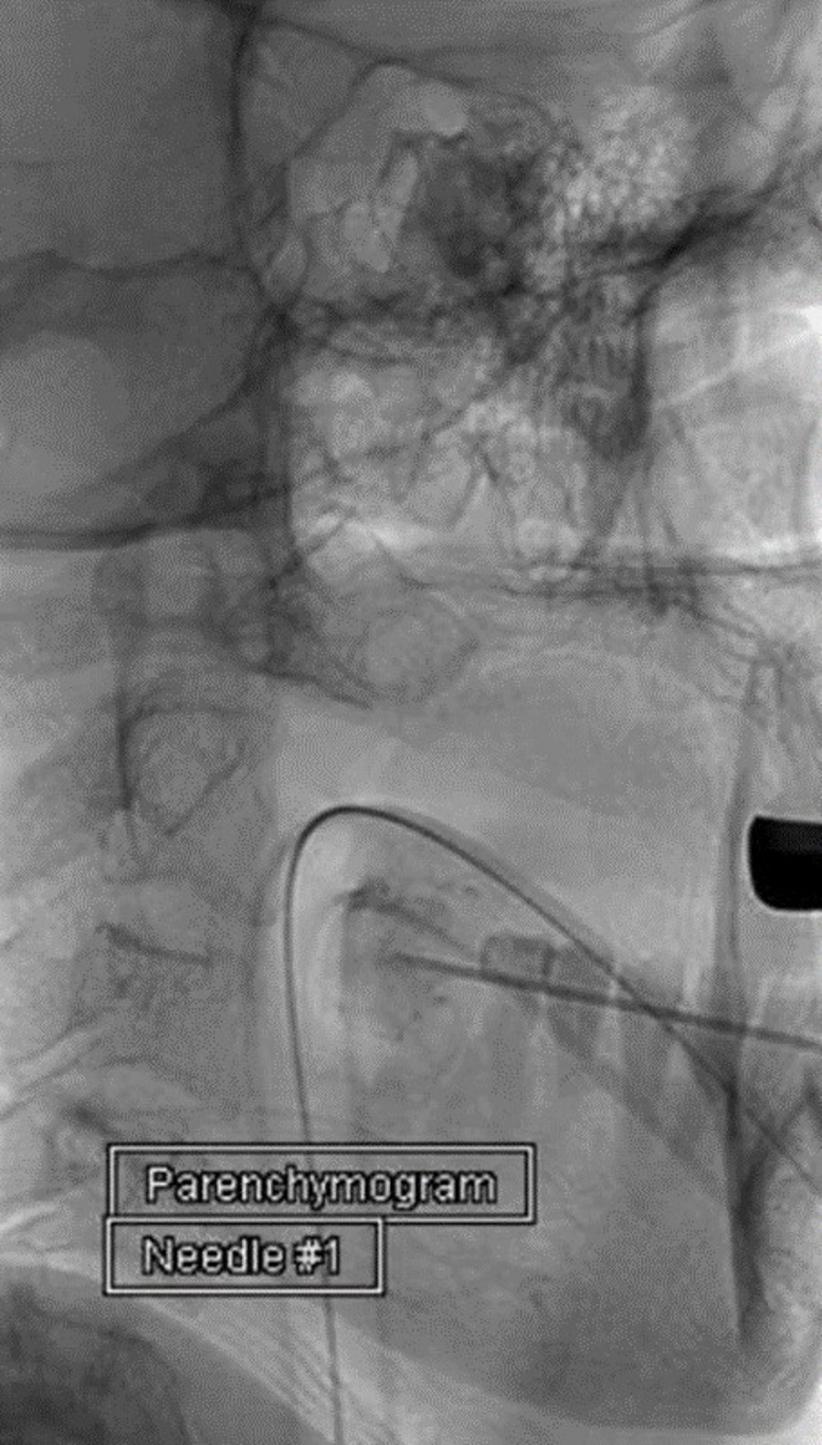
Unsubtracted parenchymogram of the lesion confirming needle position

Using fluoroscopy, a 20-gauge needle was used to puncture the lesion under fluoroscopy, and a slow steady blood flow return confirmed the appropriate location (Figure 2).

**Figure 3:**
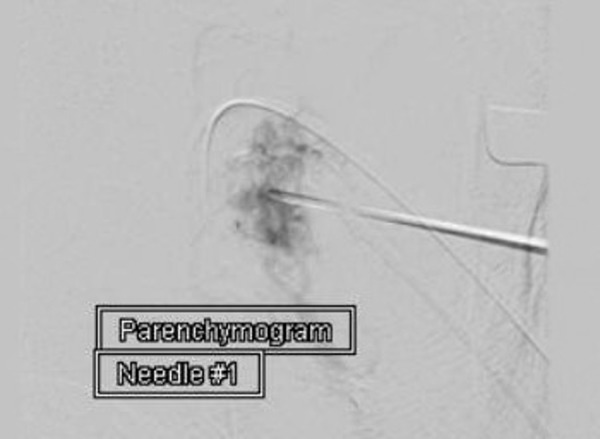
Parenchymogram in mid-arterial phase reconfirming the appropriate location of the needle

The needle was connected to tubing after being purged with DMSO. Onyx-34 was then injected under live fluoroscopic guidance after a parenchymogram was completed to serve as a baseline (Figure 3).

**Figure 4:**
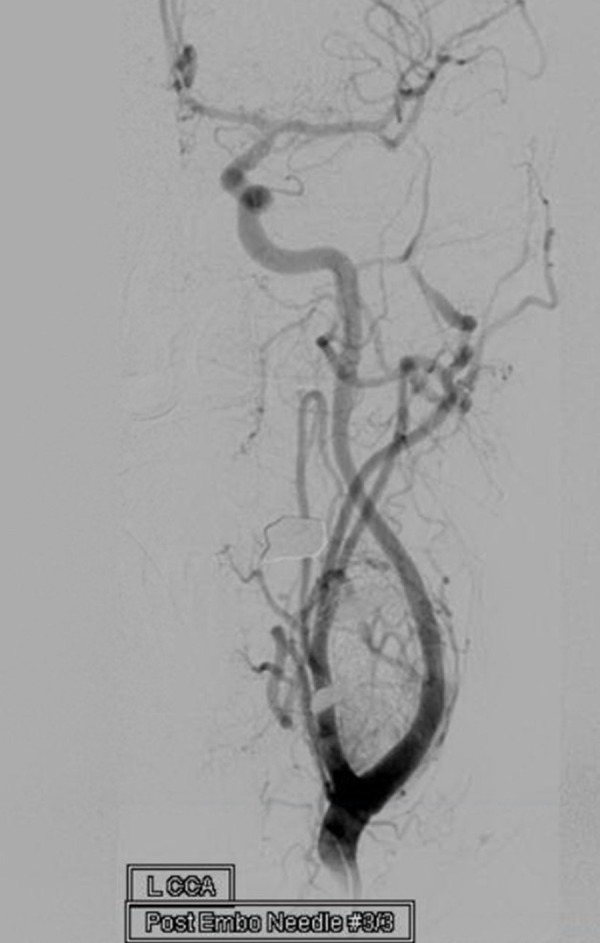
Digital subtraction angiography of the common carotid artery after treatment with Onyx revealing significant reduction in vascularity with preservation of the parent arteries.

The needle was adjusted, as previously described, to attain the targeted amount of embolization. A total of 3.5 ml of Onyx-34 was injected to achieve an approximately 80% devascularization (Figure 4).

**Figure 5:**
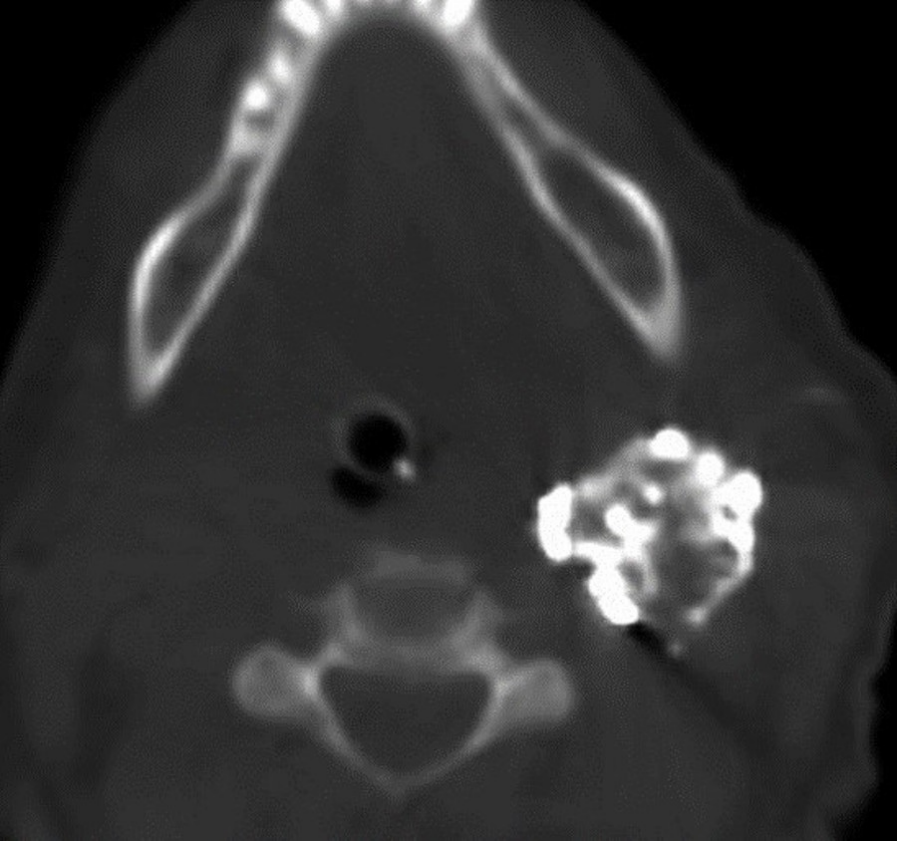
Post-treatment axial XPER CT detailing the Onyx mass within the tumor.

A post-procedure Allura XPER FD20 CT (Philips Medical Systems, Netherlands) was completed, revealing the Onyx mass (Figure 5).

## Discussion

Using preoperative embolization as an adjunct to surgery facilitates a less morbid and safer operation [11-12]. The goal of this study is to add to the growing body of literature regarding this neuro-interventional technique. Casasco, et al. first described direct devascularization of head and neck tumors in 1994 [13-14]. Prior to this, direct transarterial embolization was the favored treatment modality. Since then, there have been more widely available embolic agents. Initial reports employed n-butyl cyanoacrylate (n-BCA), but more recently, Onyx has been used with success as well [1-2, 4, 6-7, 15-16]. In almost all cases employing Onyx, by utilizing strict adherence to technique as well as aiming for near-complete devascularization, there have been few complications associated with this procedure. Major complications that can be associated with this technique can be an inadvertent migration of embolization agent, chemical toxicity, or cranial nerve palsies. In reviewing published case series regarding direct intralesional embolization, one patient developed Horner’s syndrome and lower cranial nerve palsies [16]. In most other published series, there have been no untoward events related to the direct intralesional embolization employing Onyx. It is our preference to have an objective of subtotal or near complete devascularization as the goal of intervention. In particular, we are more aggressive with the superior portion of the tumor, as this is more distant from the carotid bifurcation and cranial nerves present in the caudal aspect.  In addition, the improved safety profile may be due to characteristics inherent to Onyx. Specifically, Onyx has a more uniform and definite composition which facilitates a safer and more controlled intralesional injection. Also, direct visual feedback during injection allows the operator to cease injection immediately. Additionally, balloon occlusion of the internal carotid artery (ICA) is not necessary during intralesional therapy to safely perform direct puncture embolization therapy [1-2, 6-9, 17]. The results of available literature are summarized in Table 1. In all case series employing direct puncture therapy as an adjunct to surgical treatment, the use of Onyx facilitated resection by delineating the boundary of the tumor.

**Table 1:** Summary of studies utilizing Onyx for direct puncture of carotid body tumors or paragangliomas

Direct Puncture of Carotid Body Tumors or Paragangliomas Using Onyx						
Authors	Patients	Fluoro Time	% Devascularization	Balloon Use	Onyx Amount	Complications
Elhammady 2009	1	20 minutes	100%	No	5.0 ml	None
Wanke 2009	6	19.45 minutes	100%	Yes	4.75 ml	None
Ulrich 2009	1	N/A	100%	No	9.0 ml	None
Wiegand 2010	1	N/A	100%	Yes	20.0 ml	CN 12 and 9 edema Horner's
Ozyer 2010	1	N/A	90%	No	7.5 ml	None
Martinez-Galdamez 2011	6	N/A	>95%	No	7.0 ml	None
Shah 2012	7	23 minutes	100%	No	8.7 ml	None
Elhammady 2012	18	40 minutes	87%	No	8.4ml	None
Griandzde 2013	10	N/A	98%	No	N/A	None

## Conclusions

A review of the available literature for direct intralesional embolization of carotid body tumors reveals a safe and effective manner of tumor devascularization to facilitate open surgery. In addition, the reported complication rates from using Onyx for this technique have been minimal. Our own preliminary experience echoes and adds to the current body of literature regarding direct intralesional therapy.
